# Investigation of the association between *SLC1A3* gene polymorphisms and normal tension glaucoma

**Published:** 2011-03-25

**Authors:** Reiko Yasumura, Akira Meguro, Masao Ota, Eiichi Nomura, Riyo Uemoto, Kenji Kashiwagi, Fumihiko Mabuchi, Hiroyuki Iijima, Kazuhide Kawase, Tetsuya Yamamoto, Makoto Nakamura, Akira Negi, Takeshi Sagara, Teruo Nishida, Masaru Inatani, Hidenobu Tanihara, Makoto Aihara, Makoto Araie, Takeo Fukuchi, Haruki Abe, Tomomi Higashide, Kazuhisa Sugiyama, Takashi Kanamoto, Yoshiaki Kiuchi, Aiko Iwase, Shigeaki Ohno, Hidetoshi Inoko, Nobuhisa Mizuki

**Affiliations:** 1 Department of Ophthalmology, Yokohama City University School of Medicine, Yokohama, Kanagawa, Japan; 2 Department of Legal Medicine, Shinshu University School of Medicine, Matsumoto, Nagano, Japan; 3 Department of Ophthalmology, University of Yamanashi, Faculty of Medicine, Yamanashi, Japan; 4 Department of Ophthalmology, Gifu University Graduate School of Medicine, Gifu, Japan; 5 Department of Surgery, Division of Ophthalmology, Kobe University Graduate School of Medicine, Kobe, Hyogo, Japan; 6 Department of Ophthalmology, Yamaguchi University School of Medicine, Ube, Yamaguchi, Japan; 7 Department of Ophthalmology and Visual Science, Graduate School of Medical Sciences, Kumamoto University, Kumamoto, Japan; 8 Department of Ophthalmology, University of Tokyo School of Medicine, Tokyo, Japan; 9 Division of Ophthalmology and Visual Science, Graduated School of Medical and Dental Sciences, Niigata University, Niigata, Japan; 10 Department of Ophthalmology and Visual Science, Kanazawa University Graduate School of Medical Science, Kanazawa, Ishikawa, Japan; 11 Department of Ophthalmology and Visual Science, Graduate School of Biomedical Sciences, Hiroshima University, Hiroshima, Japan; 12 Department of Ophthalmology, Tajimi Municipal Hospital, Tajimi, Gifu, Japan; 13 Department of Ocular Inflammation and Immunology, Hokkaido University Graduate School of Medicine, Sapporo, Japan; 14 Department of Genetic Information, Division of Molecular Life Science, Tokai University School of Medicine, Isehara, Kanagawa, Japan

## Abstract

**Purpose:**

To investigate whether the solute carrier family 1, member 3 (*SLC1A3*) gene, which encodes the glutamate aspartate transporter, is associated with normal tension glaucoma (NTG) in Japanese patients.

**Methods:**

Two hundred and ninety-five Japanese patients with NTG and 518 Japanese healthy controls were recruited. Patients exhibiting comparatively early NTG onset were selected because early onset suggests that genetic factors may show stronger involvement. We genotyped 5 single-nucleotide polymorphisms (SNPs) in *SLC1A3* and assessed the allelic and genotypic diversity among cases and controls.

**Results:**

There were no statistically significant differences in the frequency of *SLC1A3* alleles and genotypes between cases and controls.

**Conclusions:**

Our study showed no association between *SLC1A3* and NTG, suggesting that the *SLC1A3* gene may not be an associated factor in NTG pathogenesis.

## Introduction

Glaucoma is characterized by the progressive degeneration of retinal ganglion cells (RGCs) and optic nerve axons, together with visual field damage, which is usually associated with elevated intraocular pressure (IOP). Primary open angle glaucoma (POAG) is the most common type of glaucoma. Normal tension glaucoma (NTG) is an important subset of POAG; while many POAG patients have high IOP [1], patients with NTG have statistically normal IOP [2-4]. NTG is more prevalent in the Asian population, particularly in the Japanese population [5-7], and recent studies in the Japanese population have reported that 92% of patients with POAG had NTG, with an IOP of 21 mmHg or less [5]. Glaucoma is generally a multifactorial disorder affected by several interacting factors, and factors other than IOP are likely to play a role in the pathogenesis of glaucomatous optic neuropathy, particularly in patients with NTG [8,9].

Glutamate is the most major excitatory neurotransmitter in the mammalian retina; excessive stimulation of the glutamatergic system has been suggested as a risk factor for the death of RGCs in glaucoma [10-15], and factors such as reduced ocular blood flow, ocular vascular dysregulation, and systemic blood pressure alterations have been investigated extensively [16-18]. The solute carrier family 1, member 3 (*SLC1A3*) gene, which is located on chromosome 5p13, encodes a glial-type, high-affinity glutamate aspartate transporter (GLAST) [19]. GLAST is tightly regulated to remove glutamate from the extracellular fluid in the retina, and is essential to keep extracellular glutamate concentrations below the neurotoxic level [15,20,21]. Therefore, it has been hypothesized that GLAST dysfunction and the resultant elevation of glutamate levels may underlie or contribute to the death of RGCs in glaucoma patients.

Based on published results, we hypothesized that *SLC1A3* may play an important role in the development of NTG. To verify this hypothesis, we performed SNP analysis of *SLC1A3* and investigated the disease susceptibility of *SLC1A3* polymorphisms in NTG patients.

## Methods

### Participants

We recruited 295 unrelated Japanese patients with NTG and 518 unrelated healthy Japanese controls at Yokohama City University, Yamanashi University, Gifu University, Kobe University, Yamaguchi University, Kumamoto University, Hokkaido University, Tokyo University, Niigata University, Kanazawa University, Hiroshima University, Tajimi Municipal Hospital, and Tokai University in Japan. The criteria used to diagnose NTG have been previously described [22]. In detail, the patients were diagnosed by the following strict inclusion criteria: the presence of glaucomatous optic neuropathy with corresponding visual field loss; normal open angle with angle width of Shaffer grade 2 or higher; absence of IOP greater than 21 mmHg on repeat measurement using Goldmann applanation tonometry without medication; and lack of pathological basis for optic nerve change upon neurologic, rhinologic, and general medical examination, including magnetic resonance imaging. Glaucomatous optic nerve abnormality was diagnosed when the vertical cup/disc ratio of the optic nerve head was 0.7 or higher, the rim width at the superior (11 to 1 h) or inferior (5 to 7 h) portion was less than or equal to 10% of the disc diameter, the difference in the vertical cup/disc ratio between eyes was 0.2 or greater, or a nerve fiber layer defect was found. Glaucomatous visual field loss was defined on a hemifield basis using reliable field data examined by the Humphrey® static visual field analyzer (HFA) C-30-2 program (Carl Zeiss Meditec, Oberkochen, Germany) according to Anderson and Patella’s criteria [23]. The hemifield was judged abnormal when the pattern deviation probability plot showed a cluster of 3 or more non-edge contiguous points having sensitivity with a probability of less than 5% in the upper or lower hemifield, and in one of these points with a probability of less than 1%.

In addition, the following inclusion and exclusion criteria were used to stringently categorize the patient groups. We excluded individuals who were diagnosed below the age of 20 years or above the age of 60 years, as well as patients for whom the refractive error was the spherical equivalent of less than - 8.0 diopters (D). In order to correct for the damaging effects of aging on the retinal ganglion (i.e. the mean deviation [MD] measured by HFA C-30-2) the selection criteria were modified based on subject age, as follows: (i) no modification if the patient was diagnosed below the age of 50 years, (ii) -10.00 dB or worse in at least one eye if the patient was diagnosed between the ages of 50 and 55 years, (iii) -15.00 dB or worse in at least one eye if the patient was diagnosed above the age of 55 years. Cases with a comparatively early onset were selected because early onset suggests stronger involvement of genetic factors. During diagnosis, patients whose refraction values had changed due to cataract surgery, refractive surgery, etc., were excluded. In cases where glaucomatous visual field loss was present in only one eye, the refraction value and glaucomatous visual field loss of the affected eye were used. In cases where glaucomatous visual field loss was present in both eyes, the refraction value and glaucomatous visual field loss of the more severely affected eye were used.

Patient age ranged between 20 and 59 years (mean age, 46.4±8.1 years); 48.1% of patients were male and 52.5% were female. The mean refraction value was –4.05±3.00 diopters (D), and the mean deviation (MD) observed in the Humphrey static visual field determination (Carl Zeiss Meditec, Oberkochen, Germany) was −9.77±8.17 dB. Subjects having certain ocular symptoms were excluded from the patient group. In regard to systemic characteristics, 20 (6.8%) patients had hypertension and 5 (1.7%) patients had diabetes mellitus; no patients had collagen vascular diseases, cardiac diseases or other systemic symptoms.

Controls were healthy volunteers from a geographic region similar to that for the NTG patients, and the control population was comparable in age and sex to the NTG patients. The subjects were disease-free, with no hypertension, diabetes mellitus, collagen vascular disease, or other diseases. Subjects with glaucoma or other ocular disorders were not included in the healthy control group. Controls had no myopia or mild myopia with refractive errors of −3.00 D or less. This study was approved by the ethics committee of each participating institute, and complied with the guidelines of the Declaration of Helsinki. All study details were explained to all patients and controls before obtaining consent for genetic screening.

### DNA and *SLC1A3* genotyping

The QIAamp DNA Blood Maxi Kit (Qiagen, Hilden, Germany) was used to collect peripheral blood lymphocytes and extract genomic DNA from peripheral blood cells. Procedures were performed under standardized conditions to prevent variation in DNA quality.

**Figure 1:**
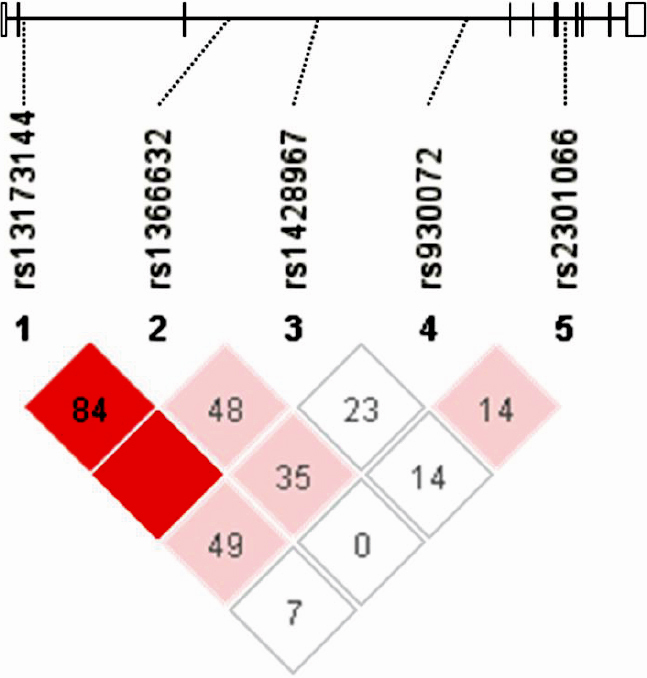
Linkage disequilibrium plot of five *SLC1A3* SNPs in 813 study participants. A schematic of *SLC1A3* is shown as a black line with black boxes representing the coding region and white boxes representing the untranslated region. The locations of the genotyped SNPs are indicated by the dotted line. The D’ value corresponding to each SNP pair is expressed as a percentage and shown within the respective square. Higher D’ values are indicated in brighter red.

We evaluated 5 single-nucleotide polymorphisms (SNPs): rs13173144, rs1366632, rs1428967, rs930072, and rs2301066. These SNPs are located within the *SLC1A3* gene, which spans over 81 kb on chromosome 5p13 and is composed of 10 exons, with minor allele frequencies >5% according to the National Center for Biotechnology Information dbSNP (Figure 1). Genotyping of all SNPs was performed by TaqMan 5′ exonuclease assay using primers supplied by ABI (Foster City, CA). The probe fluorescence signal was detected using the TaqMan Assay for Real-Time PCR (7500 Real-Time PCR System; Applied Biosystems), and following the manufacturer’s instructions.

### Statistical analysis

Hardy–Weinberg equilibrium was tested for each SNP among controls. Differences in allele frequency between case and control were assessed by χ^2^ test and Fisher’s exact test. The software Haploview 3.32 was used to compute pairwise linkage disequilibrium (LD) statistics [24]. Standardized disequilibrium (D’) was plotted. Haplotype blocks were defined according to the criteria of Gabriel et al. [25]. All p-values were derived from a 2-sided test, and p-values less than 0.05 were considered statistically significant.

## Results

Five SNPs in *SLC1A3* were genotyped. The observed and expected frequencies of each genotype for the five SNPs in the case and control participants were in Hardy–Weinberg equilibrium (data not shown), and the minor allele frequencies of all SNPs were over 5% in controls (Table 1). Figure 1 shows the strength of LD for the five SNPs in all 813 participants; no haplotype block was suggested.

**Table 1:** Allele frequencies of SNPs of *SLC1A3* among NTG patients and healthy controls.

				**Minor allele frequency**			
**dbSNP**	**Alleles (1/2)**	**Position (build 37.1)**	**Gene location**	**Cases (n=295)**	**Controls (n=518)**	**p**	**OR (95% CI)**
rs13173144	T/C	36609024	Intron	0.095	0.114	0.24	0.82 (0.58–1.14)
rs1366632	G/T	36635565	Intron	0.376	0.412	0.16	0.86 (0.70–1.06)
rs1428967	A/G	36646915	Intron	0.095	0.098	0.83	0.96 (0.68–1.36)
rs930072	C/T	36666071	Intron	0.354	0.354	1	1.00 (0.81–1.24)
rs2301066	C/T	36678275	Intron	0.361	0.393	0.20	0.87 (0.71–1.08)

1, major allele; 2, minor allele; OR, odds ratio; CI, confidence interval. Position is distance from the short arm telomere. p-Values were calculated using a χ^2^ test 2×2 contingency table.

The allele and genotype frequencies of the five SNPs in cases and controls were listed in Table 1 and Table 2, respectively. No statistically significant association was observed for any of the SNPs between cases and controls (p>0.05).

**Table 2:** Genotype frequencies of SNPs of *SLC1A3* among NTG patients and healthy controls.

		**Genotype frequency**						
		**Cases (n=295)**			**Controls (n=518)**			
**dbSNP**	**Alleles (1/2)**	**1/1**	**1/2**	**2/2**	**1/1**	**1/2**	**2/2**	**p**
rs13173144	T/C	0.817	0.176	0.007	0.780	0.212	0.008	0.46
rs1366632	G/T	0.403	0.441	0.156	0.363	0.451	0.186	0.40
rs1428967	A/G	0.814	0.183	0.003	0.807	0.189	0.004	0.97
rs930072	C/T	0.410	0.471	0.119	0.417	0.458	0.125	0.93
rs2301066	C/T	0.407	0.464	0.129	0.392	0.429	0.178	0.17

1, major allele; 2, minor allele; OR, odds ratio; CI, confidence interval. p-Values were calculated using a χ^2^ test 3×2 contingency table.

## Discussion

Glutamate transporters play a major role in maintaining physiologic extracellular glutamate levels [10]. A total of five glutamate transporters (GLAST, glutamate transporter 1 [GLT-1], excitatory amino acid carrier 1 [EAAC1], excitatory amino acid transporter 4 [EAAT4], and excitatory amino acid transporter 5 [EAAT5]) have been cloned and characterized, and GLAST (also referred to as EAAT1) and GLT-1 (EAAT2) are dominant transporter types among these [19]. Previous studies have reported the possibility of GLAST being associated with the pathogenesis of glaucoma. Naskar et al. [26] showed that glaucomatous eyes have a decreased level of GLAST. In addition, Harada et al. [27] reported that GLAST-knockout mice showed progressive RGC loss and glaucomatous optic nerve degeneration without elevated IOP, suggesting that GLAST dysfunction plays a role in NTG pathogenesis.

The aim of this study was to investigate whether polymorphisms in *SLC1A3*, the gene that encodes GLAST, affect the development of NTG. To this end, we genotyped a series of common variants located in *SLC1A3* using Japanese patients with NTG. Patients exhibiting comparatively early-onset NTG were selected for this study because early NTG onset suggests that genetic factors may show stronger involvement. Here we report a lack of association between *SLC1A3* variants and NTG in Japanese patients, suggesting that the possibility of attributing the pathogenesis of NTG to *SLC1A3* genetic variations is low.

Glaucoma is genetically heterogeneous and many genes have been reported to be associated with the development of glaucoma [18,28]. Therefore, the identification of disease susceptibility genes could provide new avenues for research aimed at clarifying the pathogenesis of glaucoma. Although the variants of *SLC1A3* encoding GLAST were not significantly associated with NTG in this study, the function of glutamate transporters still seems to have an important role in the pathogenesis of glaucoma. The other major glutamate transporter, GLT-1, has also been suggested to contribute to glaucoma pathogenesis. Martin et al. [15] demonstrated significant reductions in both GLAST and GLT-1 levels in the retina of an experimental rat glaucoma model. Sullivan et al. [29] found the evoked expression of a GLT-1 splice variant in human glaucoma and a rat glaucoma model, while glaucomatous rats exhibited no change in GLAST level. More recently, Park et al. reported that GLT-1 expression was significantly increased in cauterized eyes throughout the entire experimental period while GLAST expression remained stable during the experimental period, and surmised that GLT-1 may be a prerequisite for the maintenance of glutamate homeostasis in the retina during glaucoma development [30]. Therefore, genetic association studies of the *SLC1A2* gene, which encodes GLT-1, might be needed to clarify which transporter is the primary glutamate transporter related to glaucoma pathogenesis.

Various complex diseases often involve the integration of multiple genetic and environmental factors, and risk alleles for such diseases confer small effect sizes (odds ratio <1.5) [31], suggesting that the sample size in this study may have limited statistical power to detect risk alleles of glaucoma (a common disease with small effect sizes). Therefore, our further genetic association studies will require larger sample sizes to produce reliable data.
